# Balance Impairment in Fahr’s Disease: Mixed Signs of Parkinsonism and Cerebellar Disorder. A Case Study

**DOI:** 10.3389/fnhum.2022.832170

**Published:** 2022-03-09

**Authors:** Stefano Scarano, Viviana Rota, Luigi Tesio, Laura Perucca, Antonio Robecchi Majnardi, Antonio Caronni

**Affiliations:** ^1^Department of Biomedical Sciences for Health, Università Degli Studi di Milano, Milan, Italy; ^2^Department of Neurorehabilitation Sciences, Ospedale San Luca, Istituto Auxologico Italiano IRCCS, Milan, Italy

**Keywords:** Fahr’s disease, bilateral striopallidodentate calcinosis, balance, center of mass, posturography, gait analysis, rehabilitation

## Abstract

Fahr’s disease is a rare idiopathic degenerative disease characterized by calcifications in the brain, and has also been associated with balance impairment. However, a detailed analysis of balance in these patients has not been performed. A 69-year-old woman with Fahr’s disease presented with a long-lasting subjective imbalance. Balance was analyzed using both clinical (EquiScale, Timed Up and Go test, and Dizziness Handicap Inventory-short form) and instrumented tests (the sway of the body center of mass during quiet, perturbed, and self-perturbed stance, and the peak curvature of the center of mass during single stance while walking on a force-treadmill). The patient’s balance was normal during clinical tests and walking. However, during standing, a striking impairment in vestibular control of balance emerged. The balance behavior displayed mixed parkinsonian (e.g., slowness and reduced amplitude of movement) and cerebellar (e.g., increased sway during standing in all conditions and decomposition of movement) features, with a discrepancy between the high severity of the static and the low severity of the dynamic balance impairment. The balance impairment characteristics outlined in this study could help neurologists and physiatrists detect, stage, and treat this rare condition.

## Introduction

Fahr’s disease is a rare, idiopathic, degenerative neurological disease characterized by calcifications, typically bilateral and symmetrical, within the basal ganglia (e.g., globus pallidus, putamen, caudate nucleus), internal capsule, cerebellum (typically the dentate nucleus), thalamus, and the cerebral white matter (Hernández et al., 2012). Clinical manifestations include neurological and psychiatric features. Parkinsonism is the most common clinical manifestation of the disease, followed by cognitive impairment and cerebellar signs (Manyam, 2005). Fahr’s disease, whose actual prevalence is unknown (Mufaddel and Al-Hassani, 2014), can manifest as autosomal-dominant, familial, and sporadic forms (Manyam, 2005).

Balance impairment has been reported in patients with Fahr’s disease (Castello and Pallestrini, 1997; Wang et al., 2015; Sallì et al., 2016; Parasram et al., 2020). Nevertheless, despite its serious consequences (falls in the first place) deserving investigation in any condition, the literature does not mention any thorough examination regarding balance impairment in this particular disease. Hence, the aim of the current study was to explore in detail (with both clinical tests and instrumented measures) the balance impairment of a patient with Fahr’s disease.

## Participant

**FIGURE 1:**
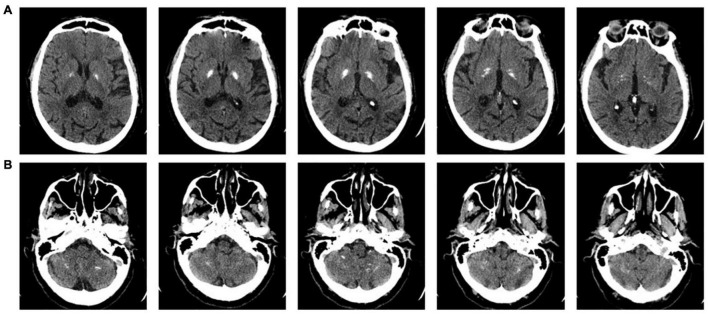
Axial sections of the present head computerized tomography (CT) scan of the patient. The upper row shows **(A)** the bilateral calcifications within the globus pallidus and cerebral white matter, and the lower row shows **(B)** the calcifications within the cerebellar hemispheres.

A 69-year-old woman with a diagnosis of Fahr’s disease was admitted to the outpatient department of a neurorehabilitation teaching hospital with the complaint of subjective balance impairment and upper limb action tremor. Fahr’s disease was diagnosed seven years earlier at a neurological university clinic. The patient’s symptoms began with perioral and upper limb tremors, followed by balance impairment, mild laryngeal dystonia, oropharyngeal dysphagia, and increasing limitations in the activities of daily living, especially manual dexterity. Head computed tomography (CT) showed calcifications within the globus pallidus, cerebral white matter, and cerebellum (Figure 1). Her medical history included hypertension and surgery for lumbar disk disease with residual chronic low back pain. An episode of paroxysmal supraventricular tachycardia occurred one year before the current assessment. Long-lasting disequilibrium with no episodes of vertigo was reported. The patient reported a few episodes of falling, with none occurring in the previous year. The patient was taking tetrabenazine (12.5 mg + 6.25 mg), primidone (31.25 mg), biperiden (0.5 mg twice a day), pregabalin (100 mg in the morning + 50 mg in the evening, prescribed for chronic low back pain), amlodipine (5 mg), olmesartan (20 mg) and bisoprolol (1.25 mg). The patient’s son also had CT evidence of calcifications in the basal ganglia and was affected by upper limb tremors.

On admission to the rehabilitation unit, the patient underwent a full medical examination by an experienced physiatrist. There was no evidence of cranial nerve impairment and, in particular, there was no nystagmus either in the primary position of gaze, with eyes’ pursuit, or with head movements. Focal strength was preserved during manual muscle testing (Ciesla et al., 2011) of the upper and lower limbs. Neuromotor examination revealed upper limb rigidity and upper limb action tremor, and manual dexterity was impaired bilaterally. Touch and vibratory sensations [as assessed with a Rydel-Seiffer 128 Hz tuning fork (Martina et al., 1998)] were preserved. The patient also complained of mild paresthesia in the L2-L3 right dermatomes, likely attributable to her back condition rather than to Fahr’s disease.

The Frontal Assessment Battery (Appollonio et al., 2005) score of 8/18 indicated impairment of executive functions. No alterations were found in the other cognitive domains (language, memory, praxis), and her total score on the Mini-Mental State Examination (Grigoletto et al., 1999) was 29/30.

The patient could perform sit-up, stance, and walking independently. The Romberg test with the feet together was negative for falling. Self-selected gait speed and step length were 0.9 m s^–1^ and 0.53 m, respectively. The patient was also independent in all home and community activities, and she did not use assistive devices.

## Materials and Methods

Self-selected walking speed and step length were assessed during an overground 10-meter walking test (average value across four repetitions).

**FIGURE 2:**
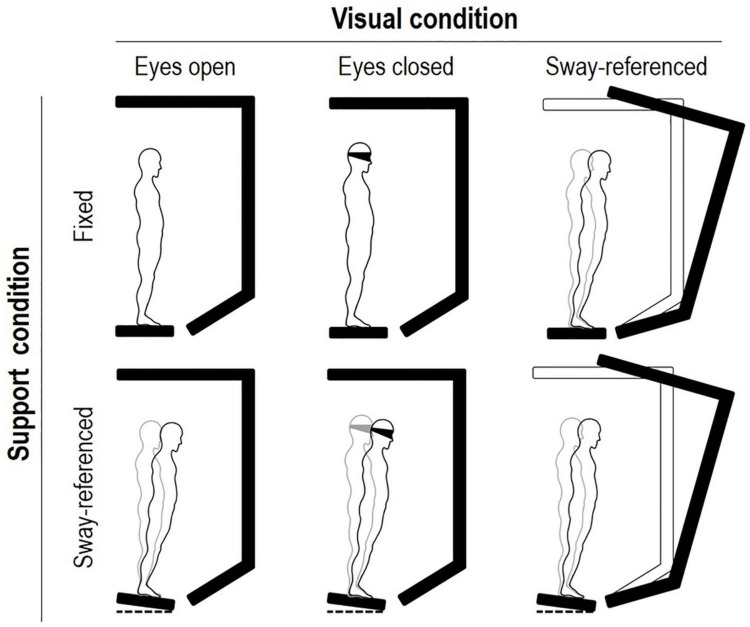
The six conditions of balance testing on the EquiTest™ system (Nashner and Peters, 1990). Conditions 1 to 3 imply a fixed support surface, conditions 4 to 6 a “sway-referenced” support oscillation. From top to bottom, conditions imply eyes open, eyes closed (or blindfolded), and sway-referenced surround oscillations, respectively. Keeping balance is progressively more difficult, from conditions 1 to 6. Three 20-s trials are requested for each condition. The subject is requested to stand still. Scores may range from 0 (stepping or falling) to 100 (no oscillations). A “composite” score is computed as the average across tests. Age-adjusted norms (95^th^ percentiles) are available for all scores. See text for details.

The sensory organization test (SOT) of the EquiTest™ system (NeuroCom^®^ International, Inc., Clackamas, OR, United States) was used to measure the center of mass (CoM) displacement during the static balance tests. The EquiTest consists of a support surface comprising a dual-force plate that can rotate in the sagittal plane, and a mobile visual surrounding of a landscape, encircling the individual. From the anteroposterior displacements of the center of pressure, dedicated algorithms (taking into account the individual’s height) estimate the displacement of the body’s CoM. Visual, proprioceptive, and vestibular balance control systems were evaluated (Figure 2). The visual surround and the force plate, separately or together, may move following the patient’s CoM anteroposterior sway (so-called sway-referenced oscillation), thus falsely signaling stability to vision (moving surround conditions) and the ankles (moving platforms conditions), respectively. The sensory systems not affected by sway-referencing should be selected as reliable systems and be able to compensate for unreliable systems. During SOT testing, the patient was asked to stand upright without wearing footwear, with foot placement standardized relative to her height and with arms relaxed at her sides. She was instructed to look forward and stand as still as possible during the trials. The patient wore a safety jacket hanging from the top of the instrument frame and was tested under six conditions, and the differences in the amount of CoM sway were measured (see Figure 2) (Nashner and Peters, 1990). Data were recorded during three trials for each condition, each lasting 20 s (see the Equitest manual, NeuroCom^®^ International Inc, 2003). A score of 0 was assigned to a trial marked as “fall” (a stepping reaction, hands touching the surround or falling and being supported by the safety jacket, within the 20-s trial), whereas a score of 100 was assigned to full stability (never achievable in practice). A cumulative 0–100 composite score is assigned to the overall test. Additional indices reflecting the functioning of specific sensory systems are also calculated (somatosensory – SOM, vision – VIS, vestibular – VEST, and visual preference – PREF; see Table 1 for details). On each test, higher scores indicated better conditions. Additional information on the SOT is available (Tesio et al., 2013; Perucca et al., 2021).

**TABLE 1:** Patient results obtained from the clinical and instrumented tests.

	Patient’s scores	Normal values
**Behavioral questionnaires**		
DHI-sf (%)	62 (± 9)	100
ES	16/16	16/16
TUG (s)	10; 8; 8	< 12.7
**Sensory Organization Test (EquiTest system)**		
Composite SOT (%)	38.0	> 68.0
SOM (%)	80.0	> 91.9
VIS (%)	62.26	> 82.6
VEST (%)	0	> 52.4
PREF (%)	97.17	> 87.5
**Limits Of Stability (Balance Master system)**		
RT (s)	0.93	< 1.50
MVL (m s^–1^)	1.80	> 2.16
EPE (%)	63.00	> 56.80
MXE (%)	84.00	> 71.60
DCL (%)	85.00	> 57.51
**CoM’s curvature peaks (treadmill walking 0.9 m s^–1^)**		
Right single stance	583.3 m^–1^	224.8–1933.1 m^–1^
Right double stance	58.9 m^–1^	79.4 – 150.0 m^–1^
Left single stance	777.9 m^–1^	340.6–1474.6 m^–1^
Left double stance	78.0 m^–1^	87.1–148.4 m^–1^

*DHI-sf, Dizziness Handicap Inventory short form; ES, EquiScale; TUG, Timed Up and Go Test; SOT, Sensory Organization Test; SOM, somatosensory; VIS, vision; VEST, vestibular control; PREF, visual preference; RT, Reaction Time; MVL, Movement Velocity (MVL); EPE, Endpoint Excursion; MXE, Maximum Excursion; DCL, Directional Control; CoM, center of mass.*

*For the TUG, the time taken to perform each of three repetitions is reported, with comparison to the upper limit of the 95% tolerance interval for elderly 60-69 years old (Steffen et al., 2002; Brusse et al., 2005).*

*In the SOT, “sensory” scores are ratios calculated as follows: SOM = equilibrium score in condition 2/equilibrium score in condition 1; VIS = conditions 4/1; VEST = conditions 5/1; PREF = conditions (3 + 6)/(2 + 5).*

*In the LOS, scores are calculated as follows: RT is the time between the “go” signal and the initiation of movement; MVL is the average speed of CoM movement between 5 and 95% of the distance to the primary endpoint; EPE is the distance traveled by the CoM on the first, ballistic attempt to reach the target, expressed in percent of LOS; MXE is the furthest distance traveled by the CoM during the trial; DCL is the ratio between the length of the straight segment from the starting to the endpoint, and the actual length of the path covered.*

*SOT and LOS scores are compared to age-matched normative data (NeuroCom^®^ International Inc, 2003), with scores considered as normal when above the 95^th^ percentile of healthy controls, for all SOT and LOS scores (with the exception of RT, for which scores are considered as normal when below the 95^th^ percentile).*

*The patient’s curvature peaks of the CoM during the stride cycle (right and left single stance phases and right and left double stance phases) are compared with the controls’ range values (mean across twenty strides for each of six healthy subjects) during treadmill walking at the same speed. The side of the double stance was defined as the side of the posterior foot.*

As clinical measures of dynamic balance, the patient underwent the Equiscale (ES) and Timed Up and Go (TUG) test. ES (Tesio et al., 1997, original in Italian) is an 8-item scale, with items scored 0, 1, or 2 (total score 0–16, the higher the better). Scores are assigned to behaviors, such as picking up an object from the floor and tandem stance, and healthy subjects achieve full scores. The ES items are administered and scored by a clinician. The TUG test (Podsiadlo and Richardson, 1991) is performed by asking the subject to sit up, walk “at a safe pace” to a target placed on the floor 3 m in front of him or her, turn, come back, and sit down. On average, community-dwelling elderly 60–69 years old complete the TUG test in 8.0 s (SD: 2.0 s) (Steffen et al., 2002), with the upper one-sided 95% tolerance interval equal to 12.7 s. To note, TUG duration > 12 s is associated with an increased risk of falling (Vieira et al., 2016).

The limits of stability (LOS) test on the Balance Master™ (NeuroCom^®^ International, Inc., Clackamas, OR, United States) was used to assess dynamic balance during voluntary movements of the trunk. The LOS is defined as the region in space through which an individual can move his or her CoM while standing on both feet without falling. The Balance Master system adopts the same algorithms for estimating the position of the body’s CoM as the EquiTest system. Through a 15-inch PC screen, the participant can see a cursor showing the instant-by-instant projection of his or her body’s CoM on the base of support. During the test, the patient was asked to stand on a static force platform and, at a “go” signal, she was requested to move the CoM to reach eight different targets. Several measures are obtained from the CoM’s path (Tesio, 2010): Reaction Time (RT), Movement Velocity (MVL), Endpoint Excursion (EPE), Maximum Excursion (MXE), and Directional Control (DCL) (NeuroCom^®^ International Inc, 2003) (see Table 1 for details).

**FIGURE 3:**
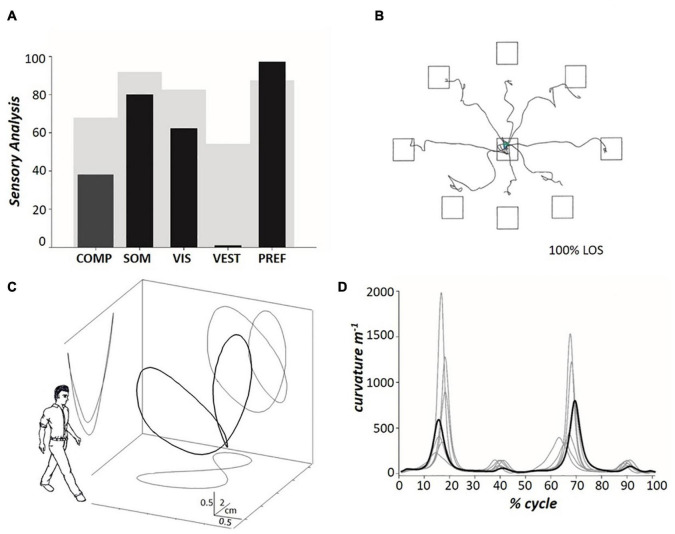
**(A)** The histograms represent, from left to right, the patient’s composite SOT (see Figure 2) and the 4 Sensory Analysis (SA) sub-scores (black columns), with comparison to normative data (gray background) for the 60–69 age group (NeuroCom^®^ International Inc, 2003). Somatosensory (SOM) represents the ratio of condition 2 to condition 1; vision (VIS) is the ratio of condition 4 to condition 1; vestibulum (VEST) is the ratio of condition 5 to condition 1; visual preference (PREF) is the ratio of conditions with unreliable vision compared to those where vision is absent, i.e., conditions (3 + 6) to (2 + 5). **(B)** The tracings represent the horizontal pathway of the patient’s CoM when leaning in the eight directions assessed during the Limit Of Stability (LOS) test. **(C)** Pathway of the patient’s CoM (the so-called “bow tie,” with projections on the sagittal, frontal and transverse planes) (Tesio et al., 2011) during treadmill walking at 0.9 m s^– 1^ (mean values across twenty subsequent strides). The outlined human form (arbitrary graphic scaling) highlights the orientation of the spatial coordinates with respect to the walking subject. **(D)** The path curvature of the patient’s CoM (bold line) (Malloggi et al., 2021) during treadmill walking at 0.9 m s^– 1^ (mean values across twenty subsequent strides), compared to the data acquired from six healthy individuals (mean ± SD age: 51.5 ± 2.5 years; range 48–54 years) walking at the same speed (light gray lines), during the stride cycle. The stride period (one stride equals two consecutive steps) was defined as the interval between two subsequent toe-offs of the posterior foot, normalized to 100 time points. Toe-off was defined as the instant at which the vertical force under the foot decreased below 30 N (Tesio and Rota, 2008). The step period was defined as the interval between the toe-off of the opposite foot and the subsequent toe-off of the corresponding foot. The side of the double stance was defined as the side of the posterior foot. Further information is provided in the text.

For the assessment of dynamic balance during walking, the patient was asked to walk on a split-belt treadmill (ADAL 3D; Médical Développement, Andrézieux-Bouthéon, France) mounted on eight force platforms (KI 9048B; Kistler, Winterthur, Switzerland); further technical details on the instrument are published elsewhere (Tesio and Rota, 2008). The treadmill was set to run in the “tied-belt” modality (i.e., with the two belts moving at the same velocity), from 0.3 m s^–1^ up to 0.9 m s^–1^ (i.e., the patient’s self-selected overground walking speed). The treadmill velocity was increased in 0.1 m s^–1^ increments, and each velocity step lasted ∼ 45 s. The patient was warned before each speed change. To ensure that the patient looked straight ahead during treadmill walking, she was asked to look at a black spot (8 cm diameter) placed ∼ 2 m in front of the treadmill at eye level on a white wall. In our experience, a visual fixation point also makes it easier for subjects to become accustomed to treadmill walking and to speed changes. No hand support was provided during the treadmill walking. Force signals were synchronized and analyzed offline using algorithms available within the SMART Software Suite (BTS Bioengineering Spa, Milan, Italy). Three-dimensional displacements of the CoM (Figure 3C) were obtained using the double integration method (Cavagna, 1975; Cavagna et al., 1983; Tesio and Rota, 2019). The path curvature (inverse of the radius of curvature) of the CoM (Figure 3D) was computed according to the Frenet–Serret formula (Tesio et al., 2011). The analysis was conducted using a custom-made MATLAB algorithm (version R2019b, MathWorks Inc., Natick, United States). For the analysis, twenty subsequent strides, acquired only at walking speed 0.9 m s^–1^ were considered. In other words, walking at speeds lower than the patient’s self-selected speed was not considered in the current study. The mean curvature peaks of the three-dimensional path of the CoM, occurring during the left-right and right-left directional changes, have been proposed as sound indices of dynamic balance during walking (Malloggi et al., 2021). The data were stored in a PC for offline analysis. Graphic representations were plotted using SigmaPlot™ (version 14.0, Systat Software Inc., San Jose, CA, United States).

Self-perception of balance impairment was assessed using the Dizziness Handicap Inventory short form (DHI-sf) (Tesio et al., 1999, validated Italian version). The DHI-sf is a 13-item questionnaire on subjective imbalance. Items were scored as 0 or 1. Representative questions are “Because of your problem, do you avoid heights?” and “Does bending over increase your problem?”. The 0–13 raw scores (the higher, the better the condition) were converted to linear 0-100 measures through Rasch analysis, provided with standard error of measurement (Tesio, 2003). The resultant scores for healthy subjects are 100%.

## Results

The entire test session lasted approximately 120 min. No side effects nor patient’s discomfort were observed.

The patient’s balance was normal on clinical tests. In particular, the patient scored 16/16 on the ES, and the mean duration of the TUG test was 8.7 s (well within the norm). However, the DHI-sf score was 62% (± 9), indicating that the patient complained of poor balance (Table 1). In particular, from the DHI-sf items, the patient affirmed to avoid heights because of her poor balance (item 6), have problems when looking up (item 1), when quickly moving her head (item 5), and when walking down a sidewalk (item 9).

The SOT score in condition 1 was slightly below the normative limit (mean score across three trials: 88.3%; normal value, nv: ≥ 90%), while scores in condition 2 (70.7%; nv: ≥ 86%), condition 3 (68.7%; nv: ≥ 80%), and condition 4 (55.0%; nv: ≥ 77%) were greatly reduced. In addition, the patient failed to complete all 20-s trials of conditions 5 and 6 (see Methods for the definition of “fall” accidents). Figure 3A represents the patient’s composite score of the SOT and the SOM, VIS, VEST, and PREF indices with comparison to normative data (60–69 years old, light gray). Composite SOT score and SOM, VIS, and VEST scores were below the normative values, with VEST displaying the most striking reduction (patient’s score: 0).

Regarding balance during trunk oscillations toward visual targets, Figure 3B shows the CoM tracings during the LOS test. The velocity of the CoM movement was reduced to below the normal limits (see Table 1). In addition, the CoM did not reach the target in five (out of eight) trials (i.e., the CoM movement was hypometric) and the CoM trajectory showed fragmentation. In contrast to the static balance tests, no falls were recorded during the LOS test.

Figures 3C,D show the tridimensional pathway and path curvature of the patient’s CoM, respectively, during treadmill walking at 0.9 m s^–1^. Figure 3D and Table 1 compare the patient data to those of six healthy individuals (range 48–54 years) walking at the same speed. The two higher peaks (i.e., the first and the third peaks, which indicate the medial-to-lateral redirection of the CoM during the single stance phase) were well within the range of the control group. The lower peaks (i.e., the second and the fourth, which occur during the double stance and indicate the CoM traveling toward the leading foot along a minimally curved trajectory) were smaller than the control peaks.

## Discussion

The current study describes a patient with Fahr’s disease with a complaint of poor balance that impacted her daily living. The posturographic assessment revealed the impairment of both static and, to a minor extent, dynamic balance, although the balance was normal on clinical examination and there was no recent history of falls. Balance impairment displayed mixed parkinsonian [e.g., slowness and reduced amplitude of movement (Postuma et al., 2015)] and cerebellar features [e.g., decomposition of movement (Holmes, 1939)].

The patient’s behavior during the EquiTest SOT may be attributable to cerebellar involvement of the disease. The impairment of static balance was severe when standing on the sway-tuned moving platform with the eyes closed (condition 5 of the EquiTest SOT, see Figure 2) or with eyes open when the visual surround was also sway-tuned (condition 6). Notably, the balance decreased even during quiet standing (condition 1). Previous studies in patients with cerebellar ataxia showed increased sway in all conditions and frequent falls in conditions 5 and 6 of the posturographic test used in this study (Gatev et al., 1996; Jacobi et al., 2015). Similar findings came from the use of triaxial accelerometers (Matsushima et al., 2015). In addition, the VEST score (which reflects the balance impairment when the vestibular system gives its maximal contribution to balance, net of the balance impairment in quiet standing) was particularly decreased in the patient, which is in agreement with studies on dynamic posturography in patients suffering from impairments of the vestibular system. Given the strict connections between the cerebellum and the vestibular nuclei (Barmack, 2003), it is not surprising that a “vestibular-like” balance impairment is found on posturographic tests in the case of cerebellar involvement. It is worth highlighting that a “vestibular” impairment on posturography does not necessarily imply primary damage to the vestibular receptors, nerves, or nuclei. By vestibular control here, it is intended the capacity to detect movements of the skull in response to inertial or gravitational forces and to generate the proper motor output to spinal motoneurons. In this broad sense, the VEST score may well be sensitive to impairments of supra-spinal (e.g., the cerebellum) structures.

In contrast to the impairment of static balance, stance during self-perturbation was mildly affected. In fact, during the LOS, the CoM trajectory actually showed reduced velocity, hypometric movements, and fragmented ataxic trajectories. However, even if parkinsonian and cerebellar signs were clearly apparent in the CoM trajectories, most LOS scores fell within the normal range. In addition, the trajectory of the CoM during the single stance phases of walking was preserved (Figure 2C), in particular, its sharp lateral-to-medial redirection at each step (i.e., the first and third peaks in Figure 2D) (Malloggi et al., 2021). On the contrary, the patient’s second and fourth peaks were lower than in the control sample. The second and fourth peaks occur in the double stance phase of walking, i.e., when the CoM is actively accelerated forward and upward by the muscular contraction and it reaches its maximum forward velocity (Tesio and Rota, 2019). It can be speculated that the reduction of the CoM’s curvature in this phase of the gait cycle helps keeping the CoM’s forward velocity as high as possible. In fact, the speed and the curvature of the movement trajectory are linked by a negative relationship (Tesio et al., 2011).

The discrepancy between the severity of impairments of static balance (the requested task is standing still), and “dynamic,” self-perturbed balance during stance and walking (the task is keeping balance while actively moving) deserves additional comments. In static balance, ballistic, high-frequency contractions of the ankle muscles are needed to maintain the CoM path within acceptable limits (Loram et al., 2005). When moving in a standing position, as when flexing the trunk, anticipatory adjustments are also at work (Morasso and Sanguineti, 2002). Additionally, anticipatory postural adjustments are also called into play during walking. During walking, the mediolateral motion displays the highest degree of instability (O’Connor and Kuo, 2009), and the lateral oscillation of the CoM runs the risk for going beyond the “no-return” inclination during the single stance. Active adjustments, likely based on anticipatory programming, allow a brisk, yet safe, lateral redirection (Tesio et al., 2009, 2010; Malloggi et al., 2021).

Therefore, why was the static balance mostly altered in this patient? This is not an entirely new finding. Previous studies have shown only weak correlations between static and dynamic balance tests in older adults (Rizzato et al., 2021), and the selective improvement of dynamic balance and gait outcomes, as opposed to the absence of significant improvement in static balance, was reported in patients with peripheral neuropathy of the lower limbs treated with physiotherapy (Caronni et al., 2019). It can be proposed that static balance, heavily based on high-frequency neural adjustments, can be more difficult to maintain, compared to dynamic balance, heavily based on low-frequency anticipatory actions and, at least in walking, profiting from passive pendulum-like oscillations and inertial stabilization.

The current study has some intrinsic limitations, the most notable being the lack of generalizability, given that a single case has been described. However, it should be highlighted that Fahr’s disease is rare. No genetic tests were performed in this case. However, Fahr’s disease displays heterogeneity in the clinical presentation even in the same pedigree and in individuals sharing the same causing mutation (Donzuso et al., 2019); hence, the genotype is possibly not relevant for the purposes of the present study.

One of the main findings of the current study is that the sensitivity of the instrumental measures of balance seems higher than that of clinical measures. Although two validated tests of balance assessment (i.e., the ES and TUG) have been used here for the clinical evaluation of dynamic balance, it cannot be excluded that there are other balance tests that are more sensitive than those used here. Indeed, there are several other balance scales besides the ES. However, compared to other clinical measures of balance [e.g., the Mini-BESTest (Franchignoni et al., 2010)], the ES investigates whether a patient completes a given balance task, regardless of *how* the patient completes it. A patient scoring 16/16 on the ES (such as the patient recruited here) successfully completed all eight balance tasks covered by the scale, including some challenging tasks such as standing with the feet in tandem position (item 8). Being able to complete a wide range of balance tasks, it seems reasonable to conclude that the patients’ balance was sound according to the clinical evaluation. However, it cannot be excluded that scales evaluating *how* patients complete the task would have revealed some balance inaccuracy. The patient’s performance on the LOS test is consistent with this view. As indicated by the good LOS scores, she successfully completed the task, but a closer inspection of the CoM trajectories indicated some signs of balance impairment.

In hindsight, balance scales including “vestibular” tasks (such as walking with head turns) (Franchignoni et al., 2010) would have been interesting in the patient’s assessment. However, the vestibular apparatus is actually stressed in at least two items of the ES (i.e., quickly turning around on the spot, and standing with the eyes closed and the head extended), and turning, a simple and physiological way to stimulate the vestibular system, is also considered in the TUG test.

Again, regarding the clinical evaluation of balance, it is also noteworthy that, while it is shown that balance was sound from the clinicians’ perspective (i.e., on clinical tests), this was not the case from the patient’s perspective (i.e., on the questionnaire of self-perceived balance impairment), as highlighted by the total score of the DHI-sf.

Regarding the gait analysis, we only analyzed the amplitude of the CoM displacement, while additional information about balance could be obtained from the CoM velocity and accelerations. In addition, gait was studied at the patient’s spontaneous speed, only, but walking at higher speeds could make an impairment of dynamic balance emerge. In this respect, it is noteworthy that walking at the maximum gait speed can make imbalance apparent even in minimally impaired patients (Caronni et al., 2020).

Lastly, regarding the study of the CoM path during walking, it can be pointed out that the patient was older than each of the six controls. Even if this represents a methodological limitation of the study, since no difference was found between the patient and the controls for this test during the single stance phases (when balance control is critical), it seems unlikely that the age difference is an issue here.

The current work has shown that instrumental measures of balance, such as those from the SOT and LOS, can provide valuable information for the clinical assessment of patients. Force platforms are a real *criterion standard* for the assessment of balance and walking. However, these instruments suffer some limitations (e.g., dedicated areas are needed, and they tend to be expensive) that could limit their wide dissemination in a clinical setting. In future studies, measures from clinical tests [e.g., the CTSIB to assess the sensory contribution to balance control (Shumway-Cook and Horak, 1986)] and low-cost instruments [e.g., inertial measurement units (Caronni et al., 2018)] could be anchored to the balance measures provided by the force platforms to facilitate the spread of instrumental measures of balance and walking among clinicians. In this line, the instrumented treadmill used here to study the CoM path during walking could be unavailable to several clinicians. In this case, the study of substitute measures of dynamic balance during walking, such as spatio-temporal or kinematic parameters of walking (Rogers et al., 2008), is clearly useful.

Thus, the case study described here seems of some interest to neurologists and physiatrists. For the clinician working in rehabilitation, it is shown that a balance assessment including clinical and instrumental tests points out varied balance impairments, whose knowledge is important for drawing up a truly patient-tailored rehabilitation program. As an example, the results of the SOT suggest specific exercise programs focused on improving balance in the absence of vision or under sensory conflicts (Tesio, 2010). In the patient described here, the exercise program should also be aimed at improving the velocity and amplitude of the voluntary displacement of the CoM, for example, during tasks requiring high dynamic balance. For the neurologists, our report shows that instrumental measures of balance can highlight and quantify subclinical balance impairment in Fahr’s disease, which could aid diagnostic purposes. Furthermore, the discrepancy between the severity of the static balance impairment and that of the dynamic balance highlights that the nervous system can compensate even a severe impairment of static balance when it comes to moving without falling. This could help develop new therapeutic approaches for improving dynamic balance once the nervous mechanisms leading to this compensation have been clarified.

## Data Availability Statement

The raw data supporting the conclusions of this article will be made available by the authors, without undue reservation.

## Ethics Statement

The studies involving human participants were reviewed and approved by Istituto Auxologico Italiano. The patients/participants provided their written informed consent to participate in this study. Written informed consent was obtained from the individual(s) for the publication of any potentially identifiable images or data included in this article.

## Author Contributions

SS and VR contributed to the design of the study. SS, VR, LP, and AR collected the data. VR processed the data and performed the analysis. SS, VR, AC, and LT analyzed the data. SS wrote the first draft of the manuscript. AC and LT revised the draft and wrote sections of the manuscript. AC revised the manuscript for intellectual content. All authors contributed to manuscript revision, read, and approved the submitted version.

## Conflict of Interest

The authors declare that the research was conducted in the absence of any commercial or financial relationships that could be construed as a potential conflict of interest.

## Publisher’s Note

All claims expressed in this article are solely those of the authors and do not necessarily represent those of their affiliated organizations, or those of the publisher, the editors and the reviewers. Any product that may be evaluated in this article, or claim that may be made by its manufacturer, is not guaranteed or endorsed by the publisher.
